# Development of nucleic acid-based vaccines against dengue and other mosquito-borne flaviviruses: the past, present, and future

**DOI:** 10.3389/fimmu.2024.1475886

**Published:** 2025-01-07

**Authors:** Muhammad Bashir Bello, Ahlam Alsaadi, Asif Naeem, Sarah A. Almahboub, Mohammad Bosaeed, Safia S. Aljedani

**Affiliations:** ^1^ Infectious Disease Research Department, King Abdullah International Medical Research Center, King Saud bin Abdulaziz University of Health Sciences, Ministry of National Guard Health Affairs, Riyadh, Saudi Arabia; ^2^ Department of Medicine, King Abdulaziz Medical City, Riyadh, Saudi Arabia

**Keywords:** denv, mosquito borne-flaviviruses, DNA vaccine, mRNA vaccine, nucleic acid based vaccines

## Abstract

Due to their widespread geographic distribution and frequent outbreaks, mosquito-borne flaviviruses, such as DENV (DENV), Zika virus (ZIKV), Japanese encephalitis virus (JEV), yellow fever virus (YFV), and West Nile virus (WNV), are considered significant global public health threats and contribute to dramatic socioeconomic imbalances worldwide. The global prevalence of these viruses is largely driven by extensive international travels and ecological disruptions that create favorable conditions for the breeding of *Aedes* and *Culex* species, the mosquito vectors responsible for the spread of these pathogens. Currently, vaccines are available for only DENV, YFV, and JEV, but these face several challenges, including safety concerns, lengthy production processes, and logistical difficulties in distribution, especially in resource-limited regions, highlighting the urgent need for innovative vaccine approaches. Nucleic acid-based platforms, including DNA and mRNA vaccines, have emerged as promising alternatives due to their ability to elicit strong immune responses, facilitate rapid development, and support scalable manufacturing. This review provides a comprehensive update on the progress of DNA and mRNA vaccine development against mosquito-borne flaviviruses, detailing early efforts and current strategies that have produced candidates with remarkable protective efficacy and strong immunogenicity in preclinical models. Furthermore, we explore future directions for advancing nucleic acid vaccine candidates, which hold transformative potential for enhancing global public health.

## 1 Introduction

Mosquito-borne flaviviruses are a collection of emerging infectious pathogens that constitute huge threats to human health globally (1). These viruses have, in recent times, caused an unprecedented increase in disease outbreaks and a dramatic expansion in their geographic distribution owing to urbanization, climate changes, international trade, and other factors that favor their continuous emergence (2, 3). Annually, not less than 400 million human infections, with several million mortalities, are recorded globally (4). Apart from their direct impact on human health, outbreaks of mosquito-borne flaviviral infections are always associated with disproportionate socio-economic imbalances, especially in developing countries, which are already confronted with other public health-related challenges (2). The most important mosquito-borne flaviviruses include dengue virus (DENV), West Nile virus (WNV), yellow fever virus (YFV), Japanese encephalitis virus (JEV), and Zika virus (ZIKV), which are all single-stranded, positive-sense enveloped RNA viruses that belong to the genus *Orthoflavivirus* in the Flaviviridae family. Their genome is approximately 10-11 kb in length and is made up of a single open reading frame that is translated into a polyprotein processed by cellular and viral proteases into 10 mature proteins (5). Three of these proteins are structural, namely, capsid (C), pre-membrane (PrM) or membrane and envelope (E), and form the core of the viral particles, while the other seven nonstructural proteins including NS1, NS2A, NS2B, NS3, NS4A, NS4B and NS5 are crucial to virus replication following infection in a susceptible host (6).

Mosquito-borne flaviviruses are transmitted by various species of mosquitoes. *Aedes aegypti* and *Ae. albopictus* mosquitoes, which are widely distributed across the tropical and subtropical areas, are responsible for the transmission of YFV, DENV, and ZIKV to humans. On the other hand, the dispersal of WNV and JEV is facilitated by *Culex* species of mosquitoes (7). Generally, Flaviviruses are maintained in an enzootic cycle between mosquitoes and mammals/avians, which act as amplifying hosts (8). Flavivirus infection in mosquitoes occurs when the mosquito ingests a blood meal containing the virus, which replicates in the insect’s midgut epithelial cells and, subsequently, the salivary gland (9), leading to the secretion of the virus in the mosquito’s saliva. Infection of a new host then occurs following mosquito bite which introduces the virus into the host, causing viraemia. While pigs and water birds function as amplifying hosts for specific flaviviruses, humans typically assume the role of dead-end hosts. This is attributed to their general inability to generate adequate viremia for the infection of other hosts, with the exception of instances involving immunocompromised individuals (10). Nevertheless, humans may serve as amplifying hosts for DENV, ZIK and YFV. Transmission may also take place through blood transfusion, sexual contact, and transplacental transmission. This transmission mode can result in abnormal gestational development in the fetus, particularly in the case of ZIKV, (11) and to a certain extent WNV.

Despite the huge public health burden of mosquito-borne flaviviruses globally, to date, no specific drugs are available to treat most of the illnesses caused by those viruses (11, 12). Consequently, vaccines stand to be the most effective countermeasures against the Flaviviruses. At present, no vaccine has been approved for the control of ZIKV and WNV (13). Noteworthy, a few vaccines have been approved to control Yellow fever, Japanese encephalitis and Dengue fever (2, 13). However, these vaccines are mainly conventional live attenuated vaccines or inactivated whole virus vaccines with serious shortcomings that significantly limit their clinical usefulness. For instance, the Dengvaxia (CYD-TDV), a tetravalent chimeric live attenuated vaccine produced by engineering yellow fever virus vaccine strain to vector the PrM and E structural proteins (PrM/E) of DENV from serotypes 1−4 (14, 15), has been shown to significantly increase the risk of cytoplasmic leakage syndrome, especially among those with no prior exposure to DENV (3, 16). As a result, the World Health Organization has discouraged the use of this vaccine in areas where Dengue fever endemicity is low (17). Similarly, 17DD, 17D-213 and 17D204 substrains are live attenuated Yellow fever vaccines developed through extensive cell culture attenuation and large-scale production in embryonated eggs (18). Although they have track records of safety and effectiveness, there are concerns that they could result in some rare side effects, such as neurologic or viscerotropic syndromes or anaphylaxis, particularly in infants, pregnant women, and immunocompromised individuals (19). More so, the development of these conventional vaccines is considerably slow and limited by the requirement of BSL-3 to produce large quantities of the vaccine virus. Thus, in order to address the expanding threats of these and several other newly emerging mosquito-borne flaviviruses, there is an urgent need for novel vaccines with remarkable effectiveness and improved safety.

With the increasing pace of genomic sequencing, reverse vaccinology approaches have evolved to rapidly identify potential protective antigens, thereby accelerating vaccine development against any pathogen at a much-reduced cost compared to the conventional approach (20). Indeed COVID-19 pandemic has shown us that emerging technologies could be used to fast track vaccine development against new public health threats in our modern society (21, 22). Some of the next-generation technologies applied to develop vaccines against mosquito-borne viruses include virus vectors, virus-like particles, engineered peptide subunits, and nucleic acid-based platforms (23). Among these platforms, nucleic acid vaccines (DNA and mRNA) stand out as the most advanced fighters against mosquito-borne flaviviruses, with several of them already advancing to various phases of clinical trials.

Although some excellent reviews on nucleic acid-based vaccines targeting specific mosquito-borne flaviviruses, DENV and ZIKV, have recently been provided (24), comprehensive overviews covering all five major mosquito-borne flaviviruses, including JEV, YFV, and WNV, are lacking scanty in the existing literature. Furthermore, available literature on modern vaccine platforms for these viruses (12, 13) often does not extensively emphasize the progress, challenges, and strategies for improving the future prospects of DNA and mRNA-based vaccines against mosquito-borne flaviviruses. Scientific challenges such as enhancing vaccine immunogenicity, ensuring broad coverage against different strains, and dealing with the complex molecular biology of flavivirus replication have further slowed progress (25, 26). Thus, there is a need for a dedicated review that focuses on the development of nucleic acid-based vaccines against all these significant pathogens, providing a detailed exploration of the past achievements, current advancements, and future directions. Our review aims to fill this gap by providing a comprehensive update on the progress and challenges associated with nucleic acid vaccine development against these rapidly emerging viruses. Additionally, we will discuss future directions for advancing these vaccines to better address global public health challenges.

## 2 Nucleic acid vaccine platform

Nucleic acid vaccines utilize the genetic material of pathogens to selectively elicit a strong immune response against the respective pathogen. These vaccines are engineered with complete instructions for producing protein antigens specific to the pathogen. Upon administration, the body’s protein-synthesizing machinery manufactures the encoded antigen, initiating an immune response. Nucleic acid vaccines may be based on either DNA or mRNA (27).

**Figure 1:**
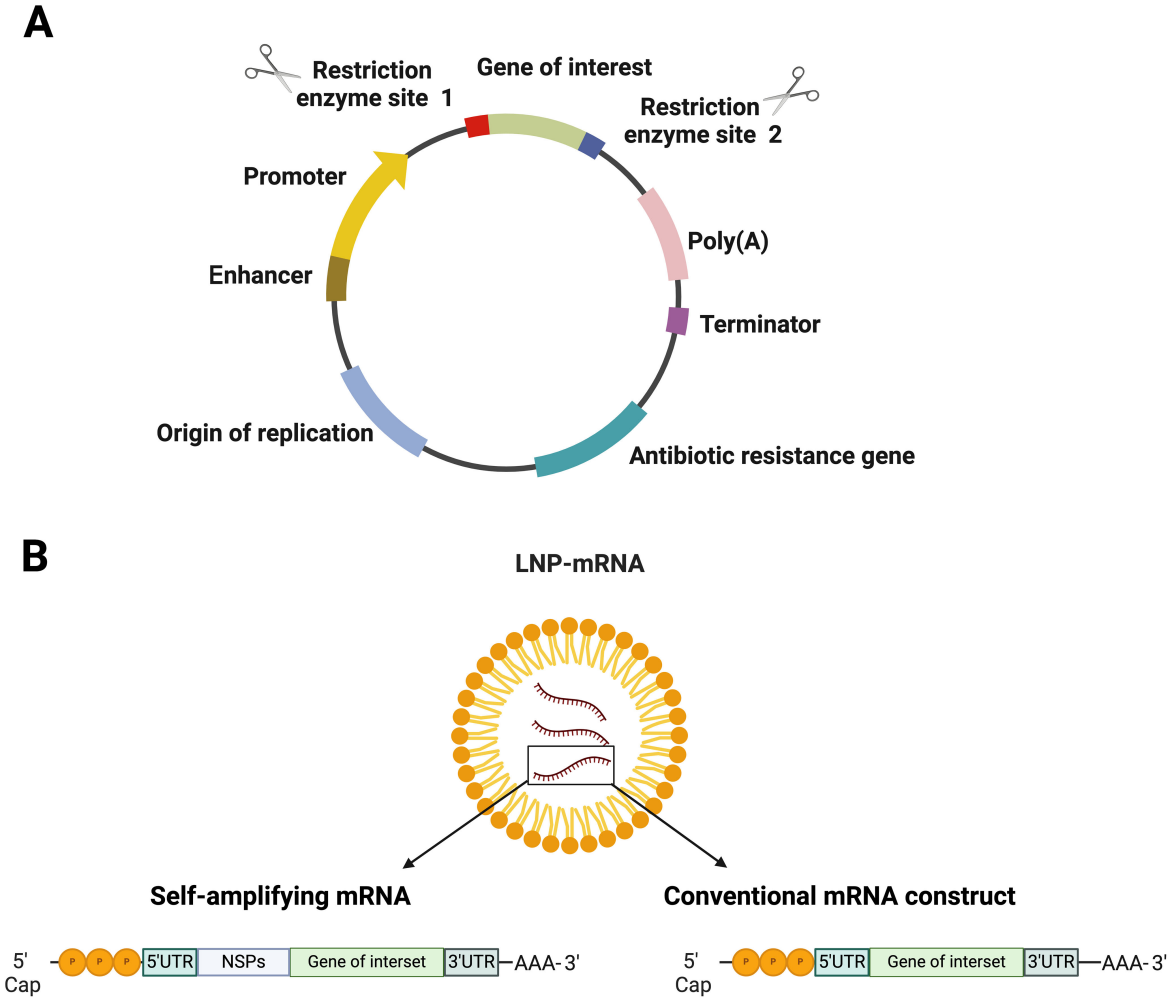
Structural elements in nucleic acid-based vaccines **(A)** DNA vaccine and **(B)** various types of mRNA vaccines encapsulated in lipid nanoparticle (LNP). Figures were created using BioRender (https://app.biorender.com).

The initial demonstration that direct *in vivo* gene delivery could trigger an immune response was by Tang et al. (28), showing an antibody response after delivering the human growth gene into mice skin using a biolistic device. Around the same time, plasmid vaccines encoding Influenza A nucleoproteins were found to elicit protective immune responses in mice, highlighting the potential of DNA vaccines (29). DNA vaccines, capable of inducing cytotoxic T cell responses, became a promising platform due to their safety, stability, and ease of manufacturing. They were developed for various infectious diseases (30, 31) and cancer (32). Plasmid DNA vaccines include key elements for efficient antigen expression, such as viral promoters (commonly the CMV promoter), the Kozak sequence for translation, and codon optimization for host-specific protein expression (33) (**Figure 1A**). Additionally, polyadenylation signals, such as the SV40 signal, enhance transgene expression. DNA vaccines can be administered through various routes like intramuscular or mucosal, where the plasmid DNA is internalized by cells and triggers immune responses via MHC pathways (34). However, despite their relative stability and ease of production, DNA vaccines generally exhibit lower immunogenicity compared to mRNA vaccines, which may necessitate multiple booster doses or the use of adjuvants to improve efficacy. The reduced immunogenicity of DNA vaccines is primarily due to their reliance on nuclear entry for transcription, an inherently inefficient process. In contrast, mRNA functions directly in the cytoplasm, enabling faster and more robust antigen expression. Safety concerns with DNA vaccines, such as the potential for genomic integration leading to mutations and the risk of inducing anti-DNA autoimmunity, further limit their widespread use (33). Consequently, only a few DNA vaccines have been approved for human use. These issues and strategies for improvement are discussed in detail in recent reviews (35–37).

**Figure 2:**
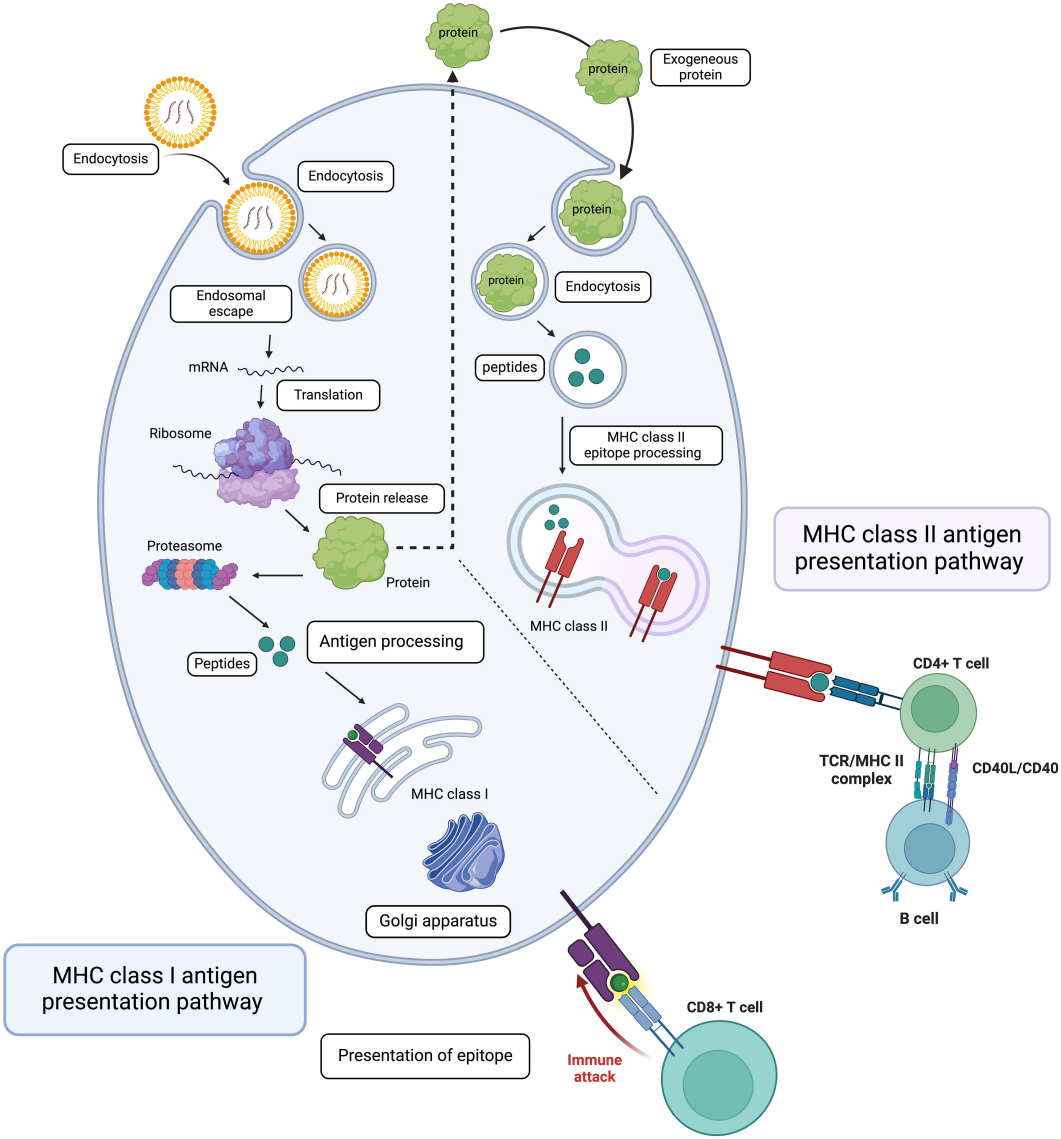
Mechanism of Action of mRNA Vaccines. This diagram illustrates the intracellular processes involved in mRNA vaccine function, starting with the transfection of mRNA into the cell and its endocytosis. The mRNA is then translated into protein by the ribosome. The synthesized proteins are processed and presented via MHC class I and II pathways, leading to the activation of the adaptive immune response. This includes the stimulation of naive CD8+ and CD4+ T lymphocytes, B lymphocytes, and the production of plasma cells, culminating in a targeted immune attack against infected cells. Figures were created using BioRender (https://app.biorender.com).

For mRNA vaccines, research dates back decades, with Wolff et al. (38) for the first time demonstrating *in vivo* protein expression in mice. This was followed by another fantastic study, which showed that diabetes insipidus could be reversed by intrahypothalamic injection of vasopressin mRNA in rats (39) (**Figure 1B**). However, mRNA technology faced challenges due to its instability and degradation before reaching target cells. Recent advancements in nanobiotechnology, particularly lipid nanoparticles (LNPs), have revolutionized mRNA vaccine delivery, allowing for robust immune responses and making mRNA vaccines critical in combating the COVID-19 pandemic (40). There are two major types of mRNA vaccines: conventional mRNA and self-replicating mRNA. Self-replicating vaccines include viral replication machinery, enabling larger antigen expression at lower doses (41). In general, the synthesis of mRNA vaccines involves the transcription of plasmid DNA templates, followed by purification to remove impurities that could cause immune reactions (42). Delivery remains a significant challenge due to mRNA’s susceptibility to degradation (43). LNPs have emerged as the leading delivery system (44), but innovations are ongoing to enhance endosomal escape and cellular uptake. Once successfully delivered into the cytoplasm of antigen-presenting cells, the mRNA construct is acted upon by the host’s translational machinery to produce the encoded protein. This process is swiftly followed by intracellular antigen processing, which culminates in the maturation of antigen-presenting cells. The matured antigen-presenting cells are characterized by the expression of co-stimulatory molecules, a response triggered by IFN-1 induction (45). Subsequently, these mature antigen-presenting cells migrate to the nearby lymph nodes. Here, they engage in close interactions with CD4+ and CD8+ cells, initiating the activation of humoral and cell-mediated immune responses through relevant major histocompatibility complex pathways. This intricate process has been extensively reviewed elsewhere (46, 47) (**Figure 2**).

It is however noteworthy that, although they are more immunogenic than DNA vaccines, mRNA vaccines are more sensitive to degradation, posing challenges in distribution, especially in regions with limited cold-chain infrastructure. While recent advancements in LNPs and nanotechnology have largely addressed these issues, their higher production complexity and cost may limit accessibility in low- and middle-income countries where mosquito-borne flaviviruses are endemic (48). The main safety concerns with mRNA vaccines include potential inflammatory reactions due to immune recognition of the mRNA itself and the risk of unintended immune responses to impurities in the vaccine. However, mRNA does not integrate into the host genome, reducing long-term risks (49).

## 3 Nucleic acid-based vaccines for flaviviruses

### 3.1 Flaviviral proteins used as vaccine antigens

**Figure 3:**
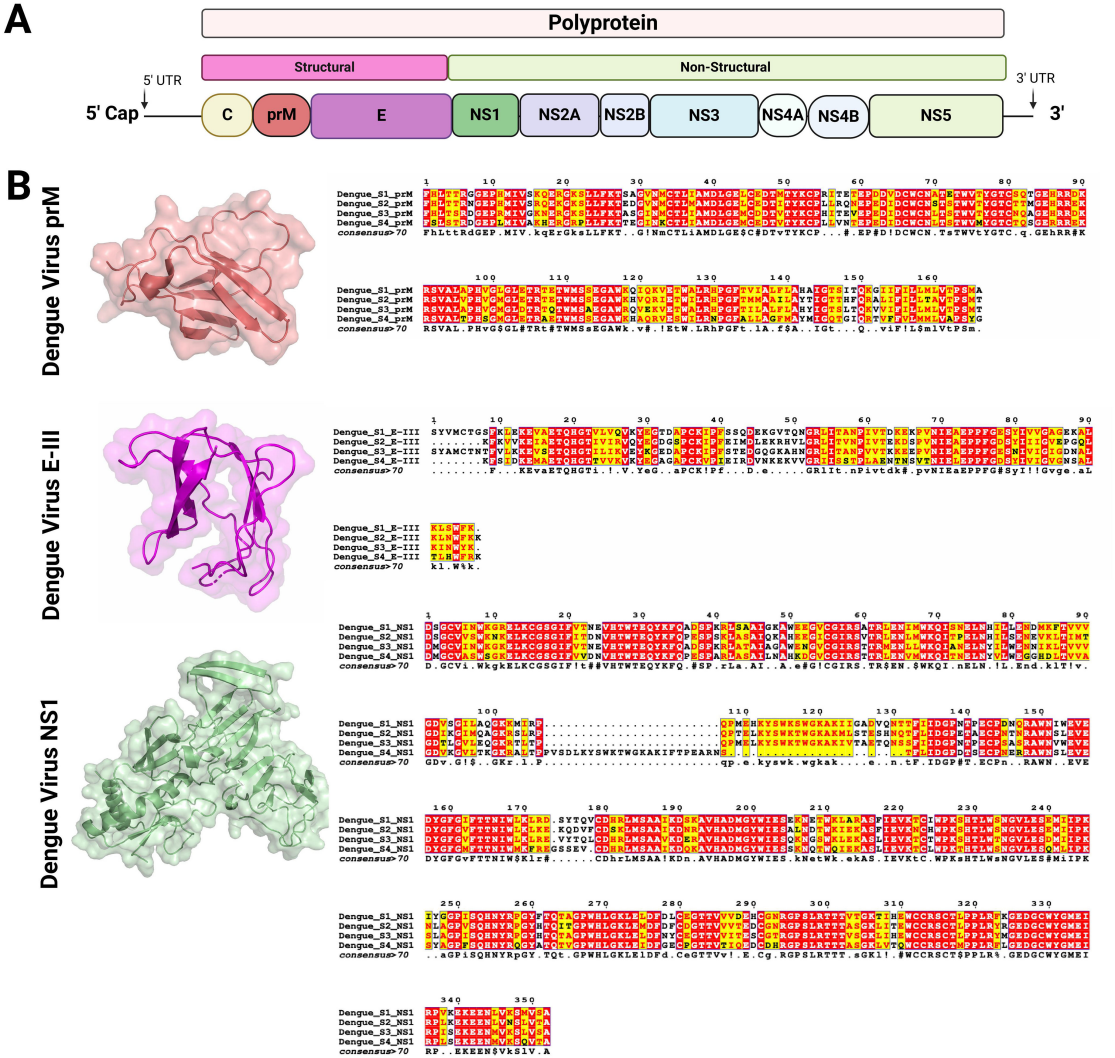
Genome organization of Flavivirus and structure of the key viral proteins used as vaccine antigens **(A)** Schematic representation of the Flavivirus genome (10-11Kb). The genome encodes a single polyprotein which is post-transcriptionally processed into three structural and seven non-structural proteins. **(B)** Structural models and sequence alignments were generated for prM, E-III, and NS1 proteins, which are used as targets for vaccine development against DENV and other mosquito-borne flaviviruses. The prM and E-III protein structures were modeled using PDB entry 8fe3 (51), while the NS1 protein structure was based on PDB entry 6weq (52). The sequence alignment highlights conserved residues across different DENV serotypes, with color coding used to indicate the level of conservation (53, 54). Figures were created using BioRender (https://app.biorender.com).

The Flaviviral genome is organized into a single open reading frame (ORF) flanked by 5’ and 3’ untranslated regions (UTRs). The ORF encodes a single polyprotein, which is cleaved by viral and host proteases into three structural proteins—capsid (C), premembrane/membrane (prM/M), and envelope (E)—and seven nonstructural proteins (NS1, NS2A, NS2B, NS3, NS4A, NS4B, NS5). The structural proteins are essential for viral particle assembly and entry into host cells, while the nonstructural proteins are involved in viral replication, immune evasion, and replication complex assembly (12) (**Figure 3A**).

In the development of novel vaccines against flaviviruses, the most commonly targeted proteins are the E, prM, and NS1 proteins (50). Although these proteins are relatively conserved across flaviviruses, their amino acid composition can vary among different viruses and even among serotypes of a single virus, such as DENV (**Figure 3B**). The E protein is crucial for viral entry into host cells and serves as the primary target for neutralizing antibodies. Structurally, the E glycoprotein consists of three domains (DI, DII, DIII), each with distinct functions (55). Domain II contains a fusion loop that facilitates membrane fusion, allowing viral RNA to enter the host cell cytoplasm. Domain III (EIII), in particular, plays a key role in receptor binding and is frequently targeted in vaccine designs to elicit neutralizing antibodies. Targeting EIII reduces the risk of antibody-dependent enhancement (ADE), a concern with dengue vaccines (56).

The prM protein acts as a chaperone during viral assembly, protecting the E protein from premature fusion before the virus matures. It ensures proper folding of the E protein, and upon maturation, prM is cleaved into the mature membrane (M) protein, making the virion infectious (57). Although prM is generally less immunogenic than the E protein, it still contributes to immune responses by promoting overall viral stability and assisting in the formation of neutralizing antibodies against the E protein. However, Antibodies generated against prM have consistently been shown to trigger ADE (58). This presents a significant challenge in the development of vaccines for dengue and other flavivirus infections, requiring careful evaluation of prM’s inclusion in vaccine formulations. While prM may play a supportive role in immune responses, its critical contribution to ADE makes it a less favorable candidate as a vaccine antigen. Despite its common use in flavivirus vaccine development efforts, we believe prM’s involvement should be reconsidered to minimize ADE-related risks. Ongoing research seeks to clarify prM’s interactions with the immune system to refine its application in vaccine design.

NS1 is a multifunctional nonstructural protein involved in viral replication and immune evasion. It exists in intracellular, membrane-associated, and secreted forms, influencing viral replication and modulating the host immune response, particularly by inhibiting complement activation (5). While NS1-targeted antibodies do not neutralize the virus directly, they can aid in eliminating infected cells and limiting viral spread. Importantly, NS1-based vaccines are less likely to induce ADE, making them an attractive alternative for dengue vaccines (59). However, NS1’s role in dengue pathogenesis must not be underestimated. First, anti NS-1 antibodies may recognize and bind to epitopes on human endothelial cells, contributing to disease pathogenesis (60). Secondly, NS-1 protein is overexpressed in severe dengue infections, functioning as a pathogen-associated molecular pattern that activates Toll-like receptor-4 on peripheral blood mononuclear cells. This activation leads to the production of pro-inflammatory cytokines and contributes to vascular leakage (61). Furthermore, anti-NS1 antibody titers are significantly higher in patients with severe dengue compared to those with mild forms of the disease. The anti-NS1 antibody repertoire also differs between severe and non-severe dengue cases, suggesting a link between specific NS1 epitopes and disease severity (62). Given these complexities, while whole NS1 is frequently used in newer vaccine designs, future strategies should prioritize targeting NS1 regions that elicit protective rather than pathogenic antibody responses to enhance vaccine safety and efficacy.

### 3.2 DNA vaccines

#### 3.2.1 DENV

The first DNA immunization against DENV was by Kochel et al. (63), showing that plasmids encoding truncated E and PrM proteins induced neutralizing antibodies in mice. Interestingly, adding CpG molecules was shown to boost this immunologic response (64). In non-human primates, full-length E and PrM plasmids led to higher antibody titers when administered intradermally (65). Studies also showed stronger immune responses with full-length E and PrM plasmids (66, 67). Moderate protective efficacy was also observed against Denv type-3 following immunization of Aotus monkeys with a plasmid DNA expressing PrM and E protein (68) even though up to 80% survival rate was observed in another study (69). Intriguingly, enhanced immunogenicity and protective efficacy were noted when a plasmid DNA expressing DENV-2 PrM/E was administered in mice via electroporation (70). The same observation was made when a bivalent vaccine consisting of pVAX1-D1ME and pVAX1-D2ME was used to immunize BalbC mice (71).

Simmons et al. (72) explored DENV DNA vaccine immunogenicity through a prime-boost strategy, using a DNA vaccine followed by protein boosters or a combination of both, showing slightly better neutralizing antibodies. Further studies (73–75) confirmed enhanced protection using DNA priming and protein/virus-vector boosting (76) also found increased antibody titers with combined DNA and protein vaccines. Adding lysosome-associated membrane protein sequences or co-administering DNA vaccines with GM-CSF improved immune responses (77, 78). Enhanced efficacy was also noted with plasmids expressing NS1 and tPA (74), though tPA didn’t always boost immunity (79). Co-administration of E and NS1 plasmids showed strong neutralizing immunity (80).

A tetravalent DNA vaccine based on domain III of the E protein or conserved epitopes from all four DENV serotypes induced long-term neutralizing immunity (81, 82) and provided strong protection against DENV type-2 (83, 84). Immunization with a mix of four plasmids expressing PrM/E genes from each serotype led to robust antibody responses post-challenge (85). Electroporation and prime-boost strategies further enhanced tetravalent immunity (86). A PrM/E tetravalent vaccine neutralized all serotypes but offered partial protection upon challenge (87). Synthetic consensus domain III of the E protein also induced strong immunity (88, 89). Combining DNA vaccines with inactivated or protein-based vaccines boosted immunogenicity (90), and priming with DNA followed by a live attenuated DENV vaccine resulted in anamnestic immune responses (Monika 91).

A DENV DNA vaccine combining PrM/E and NS-1 induced strong, long-lasting protection in mice (92), though its suitability in addressing poor immunogenicity remains uncertain. Co-expressing E and NS-1 in a bicistronic vector produced anti-E antibodies but failed to elicit NS-1 antibodies (93). Immunogenicity was lower in a PrM/E DNA vaccine compared to an inactivated DENV-4 vaccine, though 80% of mice were protected (94). Novel adjuvants have shown promise, with a lipid-based adjuvant (Vaxfectin) significantly boosting immune responses in preclinical trials (95). Higher protection was also noted when using NS1-loaded PLGA/PEG microspheres in mice (96). Co-expressing PrM/E, NS-1, and GM-CSF improved neutralizing antibodies and protection against DENV-2 (97), though caution is advised with GM-CSF due to immune suppression risks (98). A chimeric DNA vaccine substituting JEV PrM/E with DENV EDIII also generated high neutralizing antibodies and reduced infection enhancement (99).

Multi-epitope DNA vaccine that encodes conserved immunogenic HLA-restricted cytotoxic T cells epitopes derived from DENV serotype 1 was found to induce strong immune response, suggesting the possibility of using this approach in developing universal DENV vaccine (100). Similarly, Hou et al. (101) reported that that mosaic vaccines comprising of DENV serotype 1 and 2 variant epitopes could stimulate strong and broad immune responses against all four serotypes. Indeed, the strategy of fusing the consensus EDIII for each serotype with a single NSI derived one of the serotypes was proven to be efficacious in eliciting significant protective immunity in preclinical models (56). Other approaches involved the use Plasmids encoding the scFv αDEC205, or an isotype control (scFv ISO), fused to the DENV2 envelope protein domain III (EDIII) to induce neutralizing immune response (102). Furthermore, an innovative approach involving the use of adeno associated vectors to enhance the delivery of DENV DNA vaccine has recently been described (103).

#### 3.2.2 Other mosquito-borne flaviviruses

##### 3.2.2.1 Zika virus

Following the widespread ZIKV outbreaks from 2015 to 2016, Larocca et al. (104) and Dowd et al. (105) reported the development of a recombinant plasmid DNA expressing Zika pre-membrane and envelope proteins. A single administration of this vaccine provided full protection in susceptible mice when challenged with a strain of ZIKV linked to the outbreak in northeast Brazil. Same observation was made in non-human primates (106). Noteworthy, the observed protective efficacy predominantly relied on neutralizing antibody titers, since passive protection was conferred through the adoptive transfer of purified IgG from vaccinated mice and that depletion of CD4 and CD8 T lymphocytes in vaccinated mice did not diminish this protective efficacy. In another study, immunization of IFNAR-/- mice with a novel synthetic ZIKV DNA vaccine expressing PrM/E via electroporation elicited antigen-specific cellular and humoral immunity, along with neutralizing activity. This led to 100% protection of IFNAR-/- mice against infection-induced brain pathology following *in vivo* viral challenge. Moreover, passive transfer of non-human primate anti-ZIKV immune serum protected IFNAR-/- mice against subsequent viral challenge, further emphasizing the significance of immune responses targeting PrM/E in ZIKV infection (107). In addition, Yi et al. (108) showed that a DNA vaccine encoding the complete ZIKV PrM/E provides robust protection against ZIKV infection in humanized mice. Post-vaccination, the humanized DRAG mice demonstrated seroconversion, producing antibodies targeting ZIKV, as indicated by ELISA and neutralization assays. Indeed subsequent ZIKV challenge revealed markedly reduced viral loads in both human cells and serum of vaccinated animals compared to unvaccinated controls, highlighting the vaccine’s potent antiviral efficacy (108).

Maternal administration of a DNA vaccine candidate has been shown to confer specific protection against post-natal ZIKV infection in immunocompetent BALB/c mice (109). DNA immunization strategy may also offer the potential to deliver highly potent monoclonal antibodies against Zika virus for infection control in non-human primates (110). It has also been shown to reverse ZIKV-induced infertility (111) and improve negative fetal outcomes among ZIKV exposed pregnant macaques (112). Additionally, a heterologous prime-boost vaccination approach, comprising priming with a recombinant DNA vaccine followed by boosting with non-replicating vaccinia virus-based vaccines, shows promise in combatting ZIKV infection (113). In another strategy, IL-36 gamma has been shown to demonstrate excellent adjuvant properties, enhancing the protective efficacy of Zika DNA vaccine following lethal viral challenge in mouse model (114). Surprisingly, the DNA vaccine encoding the Zika EDIII domain could not provide protection against lethal viral challenge (115); despite eliciting a anti E protein antibody mediated response in the vaccinated hosts (116). This contrasts with what is known for other flaviviruses. While this result may signify the unsuitability of EDIII as a vaccine candidate against ZIKV, it is possible that the immunogenic potential of this protein may increase when used in combination with NS-1.

DNA vaccine encoding NS-1 alone was shown to elicit protective immune response in the form of reduced viremia and viral burden in tissues upon ZIKV challenge (117). Indeed, when fused to Herpes Simplex Virus (HSV) glycoprotein D (gD) protein, enhanced immunogenicity and protective efficacy were observed, further demonstrating the relevance of NS-1 protein as a Zik vaccine antigen (118). It is however crucial to highlight that prior exposure to other flaviviruses, whether through vaccination or infection, could influence the effectiveness of DNA immunization against ZIKV. For example, individuals who received the ZIK DNA vaccine (VRC5283), a plasmid encoding ZIK PrM and E, without prior exposure to flaviviruses or vaccination demonstrated limited binding ability towards various viruses in their antibody response. Conversely, those with previous flavivirus exposure displayed varying binding capability of vaccine-induced antibodies, albeit without neutralization of distantly related flaviviruses being observed (119).

##### 3.2.2.2 JEV

Lin et al. (120) demonstrated that plasmid DNA expressing JEV NS1 induces protective immunity in mice as effectively as constructs expressing PrM/E, for the first time suggesting genetic immunization as a strategy for JE infection. Although JEV E protein is key to protective immunity (121, 122), single vaccination with plasmid DNA encoding PrM/E induces low initial antibody titers, which significantly increase after viral challenge, highlighting the role of anamnestic response (123). Additionally, immunity to JEV PrM/E is long-lasting due to virus-specific memory B cells (124).

To improve the immunogenicity of a DNA vaccine expressing JEV NS-1, Chen et al. (125) co-administered it with another plasmid encoding heat shock protein 70. This approach significantly enhanced T cell proliferation and cytotoxicity, although it did not boost humoral responses. Similarly, Ashok and Rangarajan (126) showed protection against JEV challenge despite undetectable antibody titers, emphasizing the role of cell-mediated immunity (127). Other strategies include using colloidal gold particles with plasmid DNA to elicit a strong immune response (128), gene-gun delivery of chitosan-based vaccines (129), and microcapsule-encapsulated DNA vaccines, which improved Th1-Th2 responses. These findings reflect the need for integrating multiple strategies, rather than relying on individual approaches, to dramatically improve the immunogenicity of JEV DNA vaccines by effectively addressing both cellular and humoral immune responses.

A combined approach of plasmid DNA and protein-based JEV vaccines in mice led to a synergistic boost in neutralizing immune responses (130). Gene gun-assisted immunization also enhanced JEV DNA vaccine efficacy (131, 132), and the use of the Vaxfectin adjuvant further improved immunogenicity in both mice and human trials, where it was well tolerated (130, 133). A prime-boost strategy using plasmid DNA priming followed by protein-based boosting proved effective in inducing protective immunity (134, 135). Co-immunization with IL-15 also improved antibody-mediated immunity (89). Multiple strategies, such as co-administering PrM/E and GM-CSF, significantly enhanced protection against lethal JEV challenge (136, 137). However, certain cytokines could negatively impact JEV-specific responses, requiring careful evaluation of their use as adjuvants (138).

**Figure 4:**
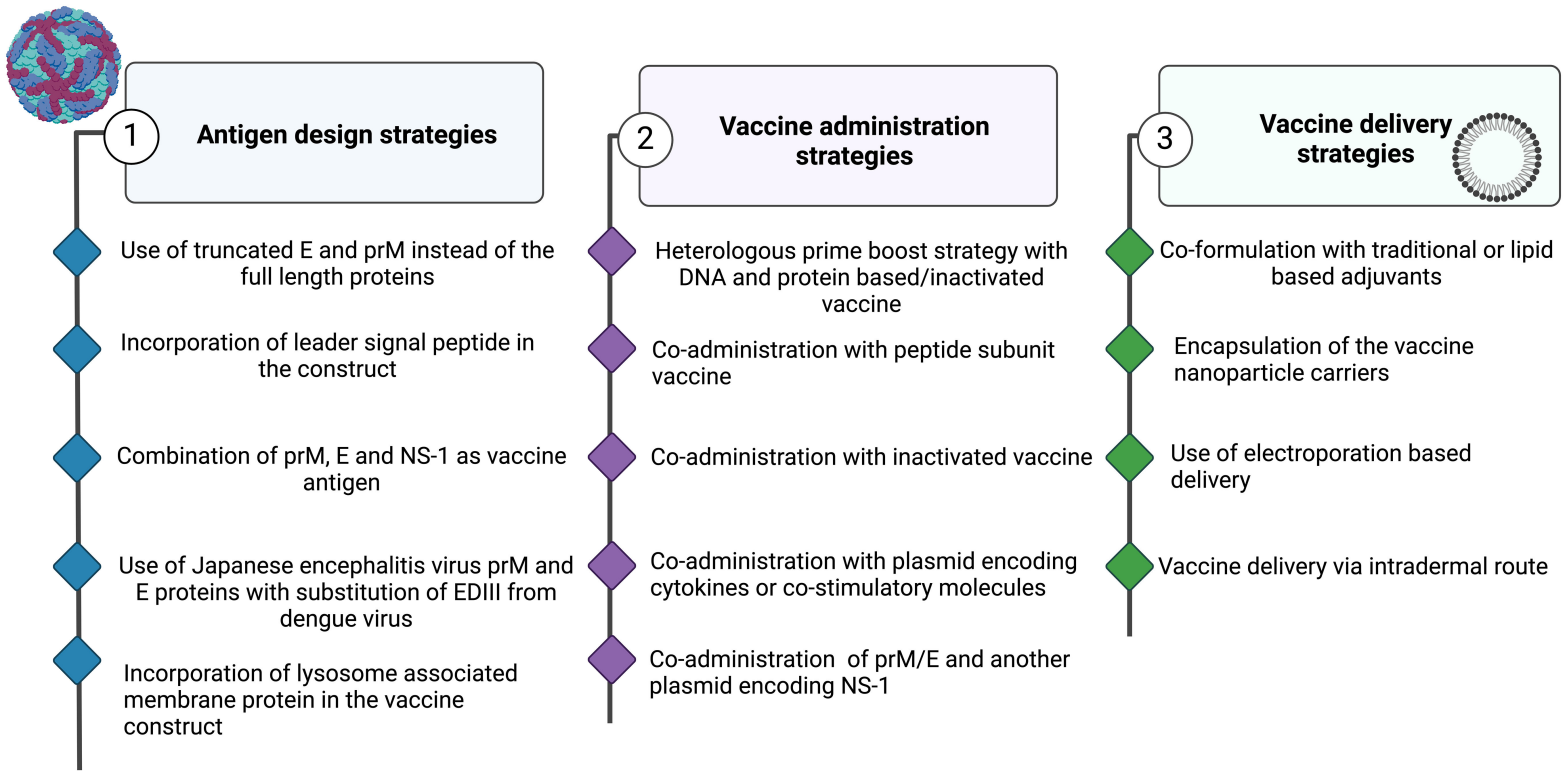
Strategies to Enhance the Immunogenicity of Nucleic Acid-Based Vaccines. Approaches Involving (1) Antigen Design, (2) Vaccine Administration, and (3) Vaccine Delivery. Figures were created using BioRender (https://app.biorender.com).

Imoto et al. (139) reported the induction of robust neutralizing antibody mediated immunity that protected against fetal mummification in pregnant sows following needle free immunization with a mixture of JEV plasmid DNA and inactivated vaccines. In another studies, immunization with the JEV DNA vaccine construct containing murine ICAM-1 gene (pICAM-1) resulted in a notable increase in the percentage of CD4(+) and T cells, high level of JEV-specific cytotoxic T lymphocyte response, and high production of T helper 1 (Th1)-type cytokines in splenic T cells (140). Thus, the strategies for improving the immunogenicity of JEV DNA vaccines are numerous, involving the rational manipulation of the vaccine antigen, use of novel adjuvants and optimal use of vaccine delivery systems (**Figure 4**).

##### 3.2.2.3 YFV

Although DNA vaccine has been developed against a variety of pathogens more than 3 decades ago, it is only recently that platform was utilized for YFV control. This might not be unconnected with the long-standing success of the existing 17D YFV vaccines. The iDNA vaccine technology represents a newly emerged vaccine strategy that combines the safety of DNA vaccine and the efficacy of live attenuated vaccines. In this approach, the entire genomic RNA of the virus is placed under the control of a eukaryotic promoter, allowing intact viral RNA to be transcribed *in vivo* following vaccination. This enables limited rounds of virus replication, as demonstrated by (141) in their study on a novel vaccine against Venezuelan equine encephalitis virus. Using this technology, Tretyakova et al. (142) showed that immunization of BalbC mice with a plasmid DNA containing the full-length 17D YFV cDNA under the transcriptional control of cytomegalovirus promoter induced robust seroconversion and virus-specific neutralizing antibodies. It was also shown to be safe in AG129 mice, genetically engineered mice that lack IFN α/β/γ receptors.

In another study, two DNA vaccine candidates encoding the full-length YFV envelope protein or the full-length YFV envelope protein fused to the lysosomal-associated membrane protein signal, LAMP-1 were reported (143). Findings revealed that vaccine constructs elicited robust T cell responses and neutralizing antibodies in C57Bl/6 and BALB/c mice. In particular, the construct with the LAMP-1 signals for enhanced MHC class II presentation generated higher interferon-gamma (IFN-γ) spot-forming cells and stronger epitope-specific responses. Furthermore, the vaccine candidates provided 100% protection against intracerebral YFV challenge in mouse models (143). This indicates that the candidates are promising and may be considered for further development in clinical studies.

##### 3.2.2.4 WNV

Several DNA vaccines have been explored for preventing WNV infection. In 2001, a DNA vaccine expressing WNV PrM proteins and E glycoprotein was developed, which provided 100% protection against WNV in mice and elicited strong neutralizing antibody responses in horses (144). A similar vaccine encoding the E glycoprotein ectodomain protected mice from WNV with a single dose, and subsequent injections of recombinant E domain DIII further boosted immunity (145). A heterologous prime-boost strategy combining DNA and inactivated vaccines led to higher neutralizing antibody levels (146). Another study using DNA priming followed by a protein boost with WNV E glycoprotein significantly improved neutralizing antibody titers and protected mice from lethal WNV challenge, although DNA alone failed to generate a measurable immune response (147).

A limited number of DNA vaccines targeting proteins other than the WNV E glycoprotein have been developed. One such vaccine focused on the C protein of the NY99 WNV strain, demonstrating strong antigen-specific humoral, Th1, and cytotoxic T-cell responses in mice (148). Hall et al. developed a DNA vaccine encoding full-length cDNA of Kunjin virus, a closely related virus to WNV, with mutation in the NS1 protein (Pro-250 to Leu). This vaccine, containing an attenuated Kunjin virus, protected mice from WNV NY99 and Kunjin viral challenges (149). Another group created a replication-defective Kunjin-based DNA vaccine that generated single-cycle viral replication, eliciting strong immune responses and virus-neutralizing antibodies in horses (150). To improve vaccine yield, capsid protein expression was enhanced by using different forms of the C protein and a stronger promoter (151).

Two DNA vaccines for WNV have undergone clinical trials. The first, VRC 302, was developed based on a licensed WNV DNA vaccine for horses and targeted the PrM and E glycoproteins of the NY99 strain. In a phase I trial (NCT00106769), it was shown to be safe, immunogenic, and capable of producing neutralizing antibodies, similar to those protective in horses (152). The second vaccine, VRC 303, an improved version of VRC 302, used a modified promoter (CMV/R) to enhance protein expression. In a phase I trial (NCT00300417), it induced strong neutralizing antibody and T-cell responses in both younger and older adults, with better T-cell responses than VRC 302 (153).

Several factors may have contributed to the lack of approved WNV vaccines despite extensive clinical trials. The unpredictable nature of outbreaks makes it challenging to design efficacy studies, as selecting appropriate geographic areas and obtaining ethical approval before outbreaks occur is logistically complex. Additionally, the disease primarily affects a subset of the population, such as individuals aged 50 or older or those with certain underlying conditions, leading to low case counts and prolonged trial enrollment (154). The high costs of vaccination programs, as indicated by cost-effectiveness models, also pose a significant barrier to large-scale implementation. To address these issues, an age- and incidence-based vaccine program could improve cost-effectiveness and reduce disease burden. Furthermore, by targeting areas with sustained high incidence, vaccination programs could ensure more consistent demand.

### 3.3 mRNA vaccines

#### 3.3.1 DENV

Unlike the DENV DNA vaccine candidates widely found in the literature, only a few mRNA vaccines are being developed against DENV. Roth et al. (59) were the pioneers in reporting the utilization of the mRNA vaccine platform against DENV infection. Their method involved the use of viral non-structural proteins, known for their conservation across all viral serotypes or their ability to induce a cross-reactive inter-serotype cell-mediated immune response. Consequently, the immunogen in their vaccine comprised a consensus 540-amino acid long multiepitope string derived from DENV-1 NS3, NS4b, and NS4. Interestingly, employing a prime-boost vaccination strategy with this modified mRNA at a low dosage, administered to human HLA class I transgenic mice, not only resulted in a robust T cell immune response but also provided significant protection against DENV-1 infection, especially when combined with a temporary inhibition of the IFN type I receptor (59). However, while the study has yielded promising data regarding protective efficacy against homologous viral challenge with DENV-1, it remains to be determined whether the immune response elicited by this vaccine is cross protective against all the DENV serotypes.

An alternative approach in the field involves formulating an mRNA vaccine that integrates PrM/E and E80—in addition to NS1 derived from DENV-2 (155). This strategy demonstrates the efficacy of E80-mRNA, whether administered independently or in conjunction with NS1-mRNA to serve as an excellent vaccine antigen. Indeed, this particular combination has been observed to trigger a robust immune response characterized by the production of potent neutralizing antibodies and T cell responses. Critically, these formulated vaccines exhibit a high level of efficacy, providing full protection against DENV-2 challenge. Notably, the inclusion of E80, either alone or in combination with NS1, has proven particularly effective in eliciting protective immunity against the DENV-2. Interestingly, vaccination with NS1-mRNA alone also stimulates antigen-specific T cell responses and the development of antigen specific antibodies, conferring partial protection against the DENV-2 virus in immunocompetent BALB/c mice (155). This suggests that the vaccine, even when focusing solely on NS1-mRNA, holds promise in conferring immunity against DENV-2 viral challenges.

In another strategy, LNP-mRNA encoding PrM and E proteins were used as a immunogen to target DENV-1 (156). The open reading frame of these fused proteins (PrM and E) was inserted downstream of various signal peptides from either Japanese encephalitis virus, IL-2, tissue plasminogen activator, or Gaucia luciferase, all previously shown to increase transgene expression (157). Mice immunization using constructs with JEV signal peptide led to robust antiviral immune responses, characterized by high levels of neutralizing antibody titers and antiviral CD4+ and CD8+ T cells. Indeed, immunocompromised AG129 mice vaccinated with the PrM/E mRNA-LNP vaccine were protected from a lethal DENV-1 challenge, demonstrating the potential of this strategy to trigger strong protective immunity against DENV as earlier asserted by (158).

As a unique approach for stimulating tetravalent immunity and simultaneously avoiding potential Antibody-Dependent Enhancement (ADE), He et al. (159) constructed a modified mRNA vaccine consisting of conserved EDIII and NS-1 from DENV-2. The vaccine antigens were placed immediately downstream of the tPA signal peptide but upstream of the C-terminal vesicular stomatitis virus G protein transmembrane and cytoplasmic domains. Findings revealed that this strategy stimulates a robust antiviral immune response that not only neutralizes all four DENV serotypes but also significantly reduces ADE (159). This demonstrates that the combination of EDIII and a conserved nonstructural protein is an attractive strategy for inducing a safe and effective immune response against DENVes (**Figure 4**).

#### 3.3.2 Other mosquito-borne flaviviruses

##### 3.3.2.1 Zika virus

Pardi et al. (160) reported that LNP-encapsulated modified mRNA encoding the ZIKV PrM/E elicited robust immune responses in animal models, with vaccinated mice and non-human primates developing high titers of neutralizing antibodies, resulting in complete protection against ZIKV challenge (161). Richner et al. (162) applied a similar approach, demonstrating that a single low-dose vaccination with a ZIKV mRNA vaccine could induce potent neutralizing antibody responses and confer protection against ZIKV infection in mice, showcasing the vaccine’s efficacy. In addition, Jagger et al. (163) reported that ZIKV mRNA vaccine encoding PrM/E elicited higher levels of antigen-specific long-lived plasma cells and memory B cells, while significantly reducing ADE in mice. Similarly, Medina-Magües et al. (164) reported that ZIKV PrM/E mRNA-LNP vaccine candidate elicited protective antibody responses in AG129 mice lacking interferon (IFN) alpha/beta/gamma receptors. More so, two-dose immunization strategy with this vaccine led to the induction of E-specific double-positive IFN-γ and TNF-α T cells in BALB/c mice. This makes it a potential candidate for further development.

Zhong et al. (165) introduced a novel approach by developing a self-amplifying mRNA (SAM) vaccine encoding ZIKV PrM/E antigens. This approach, which allows for prolonged antigen expression, induced strong immune responses in mice, signifying the potential of SAM vaccines to enhance the immunogenicity of mRNA vaccines against ZIKV. 166) explored another innovative strategy by combining mRNA encoding ZIKV PrM/E with mRNA encoding the conserved non-structural protein NS1 with the aim of eliciting both antibody-mediated and cellular immune responses. Interestingly, mice vaccinated with this combination developed high levels of neutralizing antibodies and strong T cell responses, providing significant protection against the ZIKV challenge. This shows that the inclusion of NS1 was particularly effective in enhancing the overall immunogenicity of the ZIKV mRNA vaccine candidate.

More recently, Shin et al. (167) described the development of a ZIKV mRNA vaccine encoding full-length ZIKV PrM/E proteins using porous silica nanoparticles (PPSNs) as a delivery vehicle. The vaccine was shown to elicit strong immune responses, including high levels of neutralizing antibodies and ZIKV-specific T cell responses. Furthermore, a single injection with the vaccine provided complete protection against the ZIKV challenge in C57BL/6 mice (167). This indicates that the vaccine could be a promising candidate for further clinical development and potential application against ZIKV infection. Taken together, the development of mRNA vaccine candidates against ZIKV has significantly progressed in the last few years, demonstrating promising preclinical results on safety and immunogenicity in mice models. Indeed, some candidates have already advanced to clinical trials where they demonstrate tolerability and effectiveness in healthy flavivirus seropositive and seronegative adults (168). This progress suggests a great future for mRNA vaccines as a cornerstone platform for controlling ZIKV infections. However, it is important to consider the implications of anti-ZIKV antibodies on acute DENV infections. A recent study by (Estofolete et al. (169) indicated that prior Zika infection could be a risk factor for severe dengue disease and hospitalization, though not necessarily through the widely recognized mechanism of ADE.

##### 3.3.2.2 JEV

At present, there is only one study on the development of mRNA vaccine against JEV. The vaccine candidate, similar to other mRNA vaccine candidates for flaviviruses, utilized PrM/E as antigen and was encapsulated in LNP. When used to immunize C57BL/6J mice at the dose of 15µg per mouse and boosted 3 weeks later with same dose of the vaccine, a significant increase in neutralizing antibody titer was observed, with PRNT50 reaching approximately 1:200 in vaccinated mice. More so, a strong proliferation of CD8+, but not CD4+, T cells was observed. Most importantly, a 100% protection was observed following the challenge of vaccinated mice with 1 × 10^6^ PFU JEV P3 strains 3 weeks after booster immunization (170). This indicates that mRNA vaccine is a promising platform for the development of safe and effective JEV vaccines.

##### 3.3.2.3 Yellow fever virus

Recently, a study by Medina-Magües et al. (171) found that mRNA-based vaccine candidates targeting the Yellow Fever (YF) virus elicited strong immune responses and provided significant protection in a dose dependent manner. Formulated in LNP, the vaccines were designed to express YFV PrM/E and the non-structural protein 1 (NS1). Findings revealed that vaccination with PrM/E mRNA-LNP induced high titers of neutralizing antibodies and robust T cell responses in both mice and non-human primates, leading to protection against experimental YFV challenge. Indeed, vaccinated mice not only showed reduced viral loads and minimal disease symptoms, passive transfer of their serum was able to confer protection in naive mice (171). These findings collectively indicate that mRNA vaccines encoding PrM/E and NS1 antigens have the potential to become effective YF vaccines and overcome the limitations of current vaccines.

## 4 Future trends

The development of recombinant DNA and engineered mRNA vaccines against mosquito-borne flaviviruses has recorded a great deal of progress, yet there remains a considerable journey ahead. While several candidates are currently in clinical trials (**Table 1**), further research is required to refine various aspects of these vaccines. Future efforts should concentrate on enhancing vaccine design to create more effective and safer candidates by targeting conserved regions of viral proteins, thereby reducing the risk of ADE. In this regard, utilizing a combination of the EDIII domain and various non-structural proteins (not only NS-1) as vaccine antigens could particularly be attractive.

**Table 1:** Overview of Flaviviral Nucleic acid vaccine candidates in clinical trials.

Virus	NCT #	Study Title	Status	Interventions	Sponsor	Phases	Study Design	Reference
Dengue virus	NCT00290147	Safety and Immunogenicity Study of a Dengue Virus DNA Vaccine	COMPLETED	BIOLOGICAL: D1ME100 (dengue-1 premembrane/envelope DNA vaccine)	U.S. Army Medical Research and Development Command	Phase1	Allocation: Non-randomized Intervention Model: Single group Masking: None Primary Purpose: Prevention	* Danko, et al. 2018: (133): DOI: 10.4269/ajtmh.17-0416 * Beckett et al., 2011 (172): DOI: 10.1016/j.vaccine.2010.11.050
	NCT02887482	GLS-5700 in Dengue Virus Seropositive Adults	COMPLETED	BIOLOGICAL: GLS-5700| BIOLOGICAL: Placebo	GeneOne Life Science, Inc.	Phase1	Allocation: Randomized Intervention Model: Parallel Masking: Double (Participant, Investigator) Primary Purpose: Treatment	Unpublished
	NCT01502358	Evaluation of the Safety and the Ability of a DNA Vaccine to Protect Against Dengue Disease	COMPLETED	BIOLOGICAL: Tetravalent Dengue Vaccine (TVDV) BIOLOGICAL: Tetravalent Dengue Vaccine (TVDV) with Vaxfectin¬Æ (low-dose) BIOLOGICAL: Tetravalent Dengue Vaccine TVDV with Vaxfectin¬Æ (High Dose)	U.S. Army Medical Research and Development Command	Phase1	Allocation: Non-randomized Intervention Model: Parallel Masking: None Primary Purpose: Prevention	Unpublished
	NCT03831503	A Study of INO-A002 in Healthy Dengue Virus-naive Adults	COMPLETED	BIOLOGICAL: INO-A002 DEVICE: CELLECTRA¬Æ 2000 DEVICE: Dengue Fever Antibodies (IgG)	University of Pennsylvania	Phase1	Allocation: Non-randomized Intervention Model: Sequential Masking: None Primary Purpose: Prevention	Unpublished
Zika virus	NCT03110770	VRC 705: A Zika Virus DNA Vaccine in Healthy Adults and Adolescents	COMPLETED	BIOLOGICAL: VRC-ZKADNA090-00-VP OTHER: VRC-PBSPLA043-00-VP	National Institute of Allergy and Infectious Diseases (NIAID)	Phase2	Allocation: Randomized Intervention Model: Sequential Masking: Triple (Participant, Investigator, Outcomes Assessor) Primary Purpose: Prevention	Unpublished
	NCT02840487	Safety and Immunogenicity of a Zika Virus DNA Vaccine, VRC-ZKADNA085-00-VP, in Healthy Adults	COMPLETED	BIOLOGICAL: VRC-ZKADNA085-00-VP	National Institute of Allergy and Infectious Diseases (NIAID)	Phase1	Allocation: Randomized Intervention Model: Parallel Masking: None Primary Purpose: Prevention	Gaudinski, et al., 2018 (173) DOI: 10.1016/S0140-6736(17)33105-7
	NCT02996461	VRC 320: A Phase I, Randomized Clinical Trial to Evaluate the Safety and Immunogenicity of a Zika Virus DNA Vaccine, VRC-ZKADNA090-00-VP, Administered Via Needle and Syringe or Needle-free Injector, PharmaJet, inHealthy Adults	COMPLETED	BIOLOGICAL: VRC-ZKADNA090-00-VP	National Institute of Allergy and Infectious Diseases (NIAID)	Phase1	Allocation: Randomized Intervention Model: Parallel Masking: None Primary Purpose: Prevention	Gaudinski, et al., 2018 (173) DOI: 10.1016/S0140-6736(17)33105-7
	NCT02887482	GLS-5700 in Dengue Virus Seropositive Adults	COMPLETED	BIOLOGICAL: GLS-5700 BIOLOGICAL: Placebo	GeneOne Life Science, Inc.	Phase1	Allocation: Randomized Intervention Model: Parallel Masking: Double (Participant, Investigator) Primary Purpose: Treatment	Unpublished
	NCT02809443	GLS-5700 in Healthy Volunteers	COMPLETED	BIOLOGICAL: GLS-5700	GeneOne Life Science, Inc.	Phase1	Allocation: Non Randomized Intervention Model: Parallel Masking: None Primary Purpose: Prevention	Tebas et al., 2021 (174): DOI: 10.1056/NEJMoa1708120
	NCT04917861	A Study of Zika Vaccine mRNA-1893 in Adult Participants Living in Endemic and Non-Endemic Flavivirus Areas	COMPLETED	BIOLOGICAL: mRNA-1893 BIOLOGICAL: Placebo	ModernaTX, Inc.	Phase2	Allocation: Randomized Intervention Model: PARALLEL Masking: Quadruple (Participant, Care Provider, Investigator, Outcomes Assessor) Primary Purpose: PREVENTION	Unpublished
	NCT03014089	Safety, Tolerability, and Immunogenicity of mRNA-1325 in Healthy Adult Subjects	COMPLETED	BIOLOGICAL: mRNA-1325 OTHER: Placebo	ModernaTX, Inc.	Phase1	Allocation: Randomized Intervention Model: Parallel Masking: Double (Participant, Investigator) Primary Purpose: Prevention	Unpublished
NV	NCT00300417	Phase I Study of West Nile Virus Vaccine	COMPLETED	DRUG: VRC-WNVDNA020-00-VP	National Institute of Allergy and Infectious Diseases (NIAID)	Phase1	Primary Purpose: Treatment	Ledgerwood, et al. 2011 (153) DOI: 10.1093/infdis/jir054
	NCT00106769	Vaccine to Prevent West Nile Virus Disease	COMPLETED	DRUG: VRC-WNVDNA017-00-VP	National Institute of Allergy and Infectious Diseases (NIAID)	Phase1	Primary Purpose: Treatment	Martin et al. 2007 (152) DOI: 10.1086/523650
YF	NCT01290055	Turnover of Antigen Specific Lymphocytes and Monocytes After Immunization With the 17D Yellow Fever Vaccine	COMPLETED	BIOLOGICAL: Yellow fever vaccine OTHER: Deuterium (70% enriched 2H2O) labeled water	Sri Edupuganti	Phase4	Allocation: Non-Randomize Intervention Model: Sequential Masking: None Primary Purpose: Basic Science	Unpublished
	NCT03870061	Evaluation of an Infant Immunization Encouragement Program in Nigeria	COMPLETED	BEHAVIORAL: All Babies Are Equal Initiative (conditional cash transfer program)	GiveWell	NA	Allocation: Randomized Intervention Model: Parallel Masking: None Primary Purpose: Prevention	Unpublished

To date, there are excellent candidates targeting single serotypes of DENV with remarkable preclinical efficacy. Consequently, efforts must be directed towards developing more tetravalent DENV vaccines to ensure comprehensive coverage of all circulating strains. Additionally, there is a pressing need for more mRNA vaccine candidates against all mosquito borne flaviviruses. Novel delivery platforms should also be explored to enhance immune responses and reduce dosage requirements. In this context, the development of self-amplifying mRNA vaccines, which have demonstrated enhanced antigen expression at lower doses (41), appears particularly promising. For ZIKV in particular, efforts should be focused on developing safe and effective vaccines, particularly for individuals who are pregnant or may become pregnant and those living in or traveling to Zika-endemic regions. At present, different neutralization methods are used to evaluate ZIKV neutralization titers. There is therefore the need to define universal correlates of protection based on a normalized neutralization assay, for future efficacy studies. Same applies to JEV and WNV where similar challenges in neutralization, antigen design, and correlates of protection must be addressed to improve vaccine efficacy.

## 5 Concluding remarks

It is glaringly evident that nucleic acid-based vaccines represent a promising platform for the control of mosquito-borne flaviviruses. So far, a number of candidates have been reported for DENV and other mosquito-borne flaviviruses, with several candidates in clinical trials. However, most of these candidates are DNA vaccines, with a few mRNA vaccines available for all the viruses. Currently, no mRNA vaccine for DENV is in clinical trials, while only a few are available for ZIKV. In fact, WNV does not even have a single preclinical mRNA vaccine candidate while JEV and YFV each has only a single candidate in preclinical trials. Therefore, there is a need to develop more mRNA vaccines against these viruses since mRNA vaccines have proven to be the cornerstones of modern vaccinology.

Early efforts to develop nucleic acid vaccines focused on using full PrM/E as antigens. However, it is now becoming evident that the whole envelope protein might harbor epitopes, particularly in the EDI and EDII regions, which have the tendency to elicit ADE in immunized individuals. Consequently, recent approaches are concentrating on using the EDIII domain known to be devoid of these motifs, particularly in DENV and ZIKV vaccines. New vaccine candidates are also exploring the addition of non-structural proteins to expand the breadth of the immune response to include cell-mediated immunity, which plays a crucial role in viral clearance. Furthermore, novel adjuvants, including various cytokines, are being explored to improve the immunogenicity of new vaccine candidates. Various delivery systems, including gene gun biolistics, have also been explored. Taken together, to effectively control mosquito-borne flaviviruses using either DNA or mRNA vaccine platforms, there is a need for a combination of multiple strategies encompassing rational antigen design, the use of novel adjuvants, and the careful selection of delivery systems.
